# Electroencephalogram-based multimodal attention level classification using deep learning techniques

**DOI:** 10.3389/fnhum.2026.1791677

**Published:** 2026-03-27

**Authors:** Yi Zhong, Zhenyu Wang, Xi Zhao, Tianheng Xu, Ting Zhou, Honglin Hu

**Affiliations:** 1 Shanghai Advanced Research Institute, Chinese Academy of Sciences, Shanghai, China; 2 University of Chinese Academy of Sciences, Beijing, China; 3 School of Microelectronics, Shanghai University, Shanghai, China; 4 Shanghai Frontier Innovation Research Institute, Shanghai, China; 5 ShanghaiTech University, Shanghai, China

**Keywords:** attention, BCI, deep learning, ECG, electroencephalography, EOG, multimodal

## Abstract

This study aims to develop a novel attention level prediction method using a multimodal brain-computer interface system that integrates electroencephalogram (EEG), electrocardiogram (ECG), and electrooculogram (EOG) signals to enhance prediction accuracy and robustness. We propose the Multi-Feature Enhanced Attention Network (MEAN), which leverages the complementary strengths of these signals: EEG provides insights into brain electrical activity, ECG captures heart rate variability to reflect emotional and cognitive states, and EOG records eye movements for contextual attention level information. The model is designed to address the limitations of single-modality signals, such as noise susceptibility and limited information range. Experimental results demonstrate that MEAN achieves an average accuracy of 0.9524, outperforming traditional models. The model exhibits superior adaptability, particularly in handling EEG and multimodal data, and shows enhanced predictive performance compared to existing approaches. In conclusion, the proposed MEAN model effectively integrates multimodal physiological signals to improve attention level prediction, offering a robust and accurate solution for applications requiring attention level monitoring. This research provides a foundation for advancing applications in education, work efficiency assessment, and cognitive enhancement technologies, highlighting the potential of multimodal approaches for understanding and predicting attention states.

## 1 Introduction

Attention is a cognitive focusing process that accompanies mental activities such as thinking and imagination. With advancements in science and technology, human roles in various tasks have increasingly shifted toward passive observation (Acı et al., 2019), particularly in high-precision and high-importance domains. Examples include controlling automated systems or robots and real-time metrics monitoring in specialized fields. The workers' attention level significantly affects the quality of their work. Currently, attention levels are primarily assessed through subjective judgments by observers. However, subjective judgments often fail to detect covert lapses in attention level and are subject to individual differences. Continuous real-time monitoring also incurs additional labor costs and may disrupt the normal workflow of subjects.

Moreover, since the outbreak of the COVID-19 pandemic, many countries have implemented lockdowns and social distancing measures, leading to the closure of schools, training institutions, and higher education establishments. Concurrently, advancements in computer technology and the internet have rapidly popularized online work and education (Lin and Kao, 2018). Compared to in-person teaching, online modalities have undeniably increased the difficulty of monitoring (Pokhrel and Chhetri, 2021), with numerous instances of decreased learning and work efficiency due to lack of attention being widely observed.

Brain-computer interfaces (BCI) establish a direct communication channel between the brain and computers or external devices through electroencephalogram (EEG) signals (Hu H. et al., 2024). EEG signals are crucial indicators of brain activity (Codispoti et al., 2009), originating from the electrical activity of neurons within the brain. Each neuron connects with other neurons through electrochemical processes, forming complex neural networks. When neurons receive chemical signals from other neurons, they generate minute electrical currents. These currents are transmitted and accumulated between neurons, and when they reach a certain intensity and frequency, they produce measurable brain waves (Niedermeyer, 2011). Consequently, brain waves record the coordinated activity of neuronal populations and reflect the information processing and transmission states of different brain regions. EEG signals are typically measured by electrodes placed on the scalp, which capture the faint electrical signals emitted by neuronal populations. These signals can be categorized into frequency bands such as α, β, γ, and θ waves, each corresponding to different brain activity states (Teplan, 2002). For instance, α waves are generally associated with relaxation and resting states, while β waves are related to focused attention and cognitive activity. The characteristics of these waveforms aid researchers in understanding the mechanisms underlying mental, emotional, and behavioral performance. Modern EEG technology enables researchers to explore and elucidate the relationship between EEG signals and specific behaviors or disease states. This method is not only crucial in clinical neuroscience but also holds broad application prospects in cognitive neuroscience, psychology, and neuroengineering.

Many studies, such as the work of Huang et al. (2024), have shown that EEG signals can provide real-time indications of changes in attention levels (Khedher et al., 2019). Research on attention prediction using EEG has made considerable progress. For example, Li et al. developed a real-time attention recognition BCI system based on KNN, achieving an average accuracy of 57.03% (Li et al., 2011). Belle et al. (2012) performed binary classification of attention levels using Electrocardiogram(ECG) and EEG, achieving accuracies of 77% and 86%, respectively. Subsequent studies have seen Support Vector Machines become a mainstream method for attention prediction. Based on SVMs, Ke et al. (2014) achieved a maximum three-class accuracy of 85.24%, Djamal et al. (2016) achieved a maximum binary classification accuracy of 83.0%, Alirezaei and Sardouie (2017) reached 92.8% in binary classification, and Acı et al. (2019) achieved an average three-class accuracy of 91.72%.

Although experimental methods to induce different attention levels vary among studies, the accuracy and reliability of EEG in attention level prediction have been gradually improved, with final three-class and binary classification accuracies stabilizing above 80%. Various technical methods, including KNN, SVM, Convolutional Neural Networks, and Long Short-Term Memory Networks, have been employed to address different data characteristics and task requirements. SVMs are frequently used in related research due to their accuracy. Recent studies have increasingly focused on deep learning to optimize prediction algorithms and enhance practical applicability. For instance, Jeong and Jeong (2020) investigated the potential of in-ear EEG signals to distinguish attention levels for potential applications using portable earphone-like EEG devices. Toa et al. (2021) proposed a Convolutional Attention Memory Network, demonstrating its advantages over other deep learning networks. Zennifa and Iramina (2019) used EEG, ECG, and Near-Infrared Spectroscopy (NIRS) for attention level prediction and introduced a new attention detection marker based on blink rate and pupil size measurements. Al-Nafjan and Aldayel (2022) continued to optimize and refine algorithms, while Gupta et al. (2024) incorporated facial expression information with EEG to predict students' cognitive behavior throughout the e-learning process. Nguyen et al. (2022) not only analyzed attention using the relative power spectrum of EEG signals but also proposed a new experimental device to stimulate and assess attention effectiveness through motion-induced blindness. In studies conducted within the past year on attention and fatigue, Lee et al. (2024) achieved an accuracy of 88.01% in predicting pilot fatigue in a three-class classification task. Similarly, Peng et al. (2024) developed a spatiotemporal convolutional neural network (STCNN), which, by extracting temporal, frequency, and spatial features from EEG signals, achieved a three-class classification accuracy of 87.55%. Hu F. et al. (2024) proposed a multi-branch deep learning network (STFN-BRPS) to capture the spatiotemporal features of EEG signals, achieving a three-class classification accuracy of 92.43%. Xu et al. made contributions to cross-subject attention level prediction by using Riemann geometry methods in EEG signal processing (Xu G. et al., 2023, Xu et al., 2024).

Nevertheless, relying solely on EEG signals for attention level prediction still presents challenges, such as significant individual differences among subjects, which increases prediction complexity and uncertainty. Additionally, EEG signals can be affected by noise and provide relatively limited information, leaving room for improvement in model robustness, accuracy, and temporal sensitivity. Related works, including research by Zhang et al. (2023) and Fujiwara et al. (2018), have demonstrated that introducing multimodal parameters such as Electrooculogram(EOG) and ECG can significantly enhance prediction model accuracy without markedly increasing subject burden. Through optimized signal processing and feature extraction techniques, along with comprehensive multimodal data analysis, this study presents a novel predictive model, the Multifeature Enhancement Attention Network(MEAN). Incorporating multimodal data, including EEG, ECG, and EOG, MEAN demonstrates superior adaptability and performance in within-subject analysis compared to other models. It should be noted that, as this model employs a deep learning-based Attention mechanism to predict attention levels, to avoid conceptual confusion, the terms “Attention Module” (or “Attention mechanism”) and “Attention Level” (or “Attention state”) will be used hereafter to refer to the model's attention component and the human attention state to be measured, respectively.

The main innovations and contributions of this paper are as follows:

- Proposal of a novel attention level prediction method utilizing a multimodal BCI system that integrates EEG, ECG, and EOG signals. This approach addresses the limitations of single-modality signals, such as EEG, by mitigating noise susceptibility and expanding the information scope, thereby significantly enhancing prediction robustness and accuracy.
- Design and implementation of the Multifeature Enhanced Attention Network. MEAN effectively leverages the complementary strengths of EEG, ECG, and EOG: EEG captures neural electrical activity, ECG reflects cognitive-emotional states via heart rate variability, and EOG provides contextual attention level information through eye movements, constructing a more comprehensive representation of attentional states.
- Experimental validation demonstrating the superior performance of the MEAN model, achieving an average accuracy of 0.9524. The model outperforms traditional methods and existing models, exhibiting enhanced adaptability and predictive capability, particularly in processing EEG and multimodal data.

## 2 Materials and methods

### 2.1 Data acquisition

**Figure 1:**
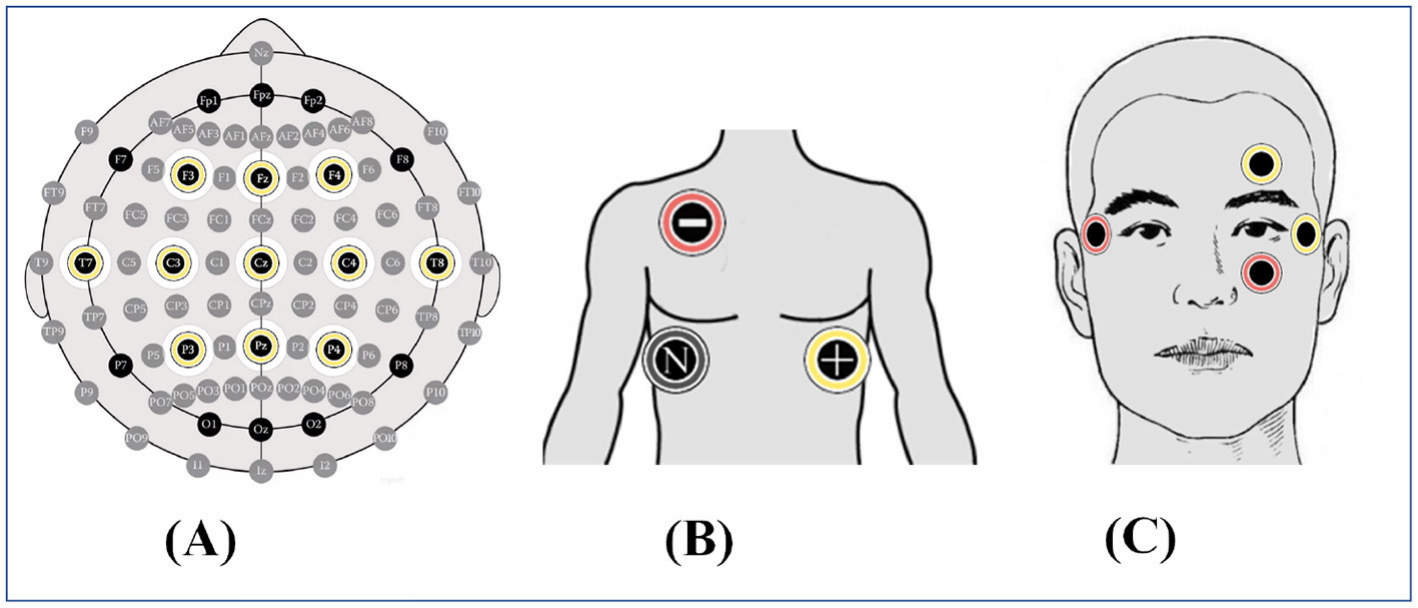
Specific locations of EEG, ECG, and EOG electrodes used in the experiment. **(A)** EEG **(B)** ECG **(C)** EOG.

**Figure 2:**
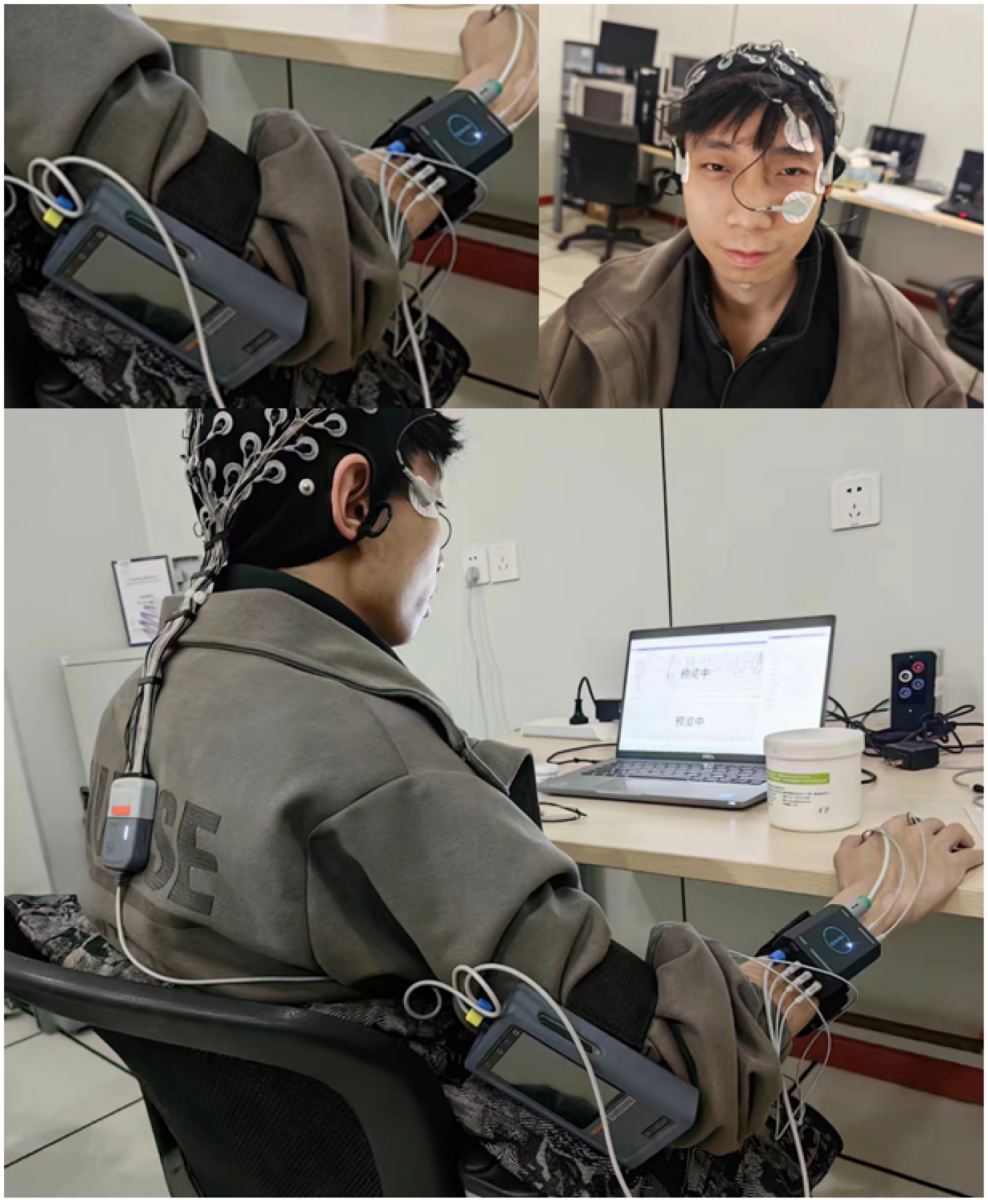
Photograph of the subject during data acquisition (the subjects have permitted the use of their image).

This study utilized the Neurohub multimodal data terminal (Huang et al., 2017) (Neuracle, Inc.) for data acquisition. This portable, wearable EEG device is specifically designed to meet the needs of neurophysiological research, enabling real-time collection of EEG, EOG, and ECG data with automatic alignment. Additionally, it can simultaneously collect heart rate and blood oxygen data. It should be noted that the raw EEG, EOG, and ECG signals were all recorded at a sampling rate of 1,000 Hz by the Neurohub system. From these signals, heart rate is a derived feature calculated from the raw ECG, while blood oxygen data is acquired via a separate finger-clip sensor with a sampling rate of 80 Hz. During subsequent preprocessing, this blood oxygen signal is synchronized to the same sampling frequency as the other physiological signals after downsampling. In this experiment, EEG data were recorded using the F3-Fz-F4-C3-Cz-C4-T7-T8-P3-Pz-P4 channels based on the 10-10 international system, totaling 11 electrodes. ECG signals were collected using adhesive electrodes, with the positive electrode placed at the V5 position of the ECG leads, the negative electrode positioned at one-third of the distance from the right clavicular notch, and the ground electrode located at the right anterior axillary line, between the fifth and sixth ribs. The device automatically generated an ECG signal channel based on these three electrodes. EOG electrodes were positioned on the EEG cap, with two channels each for horizontal and vertical eye movements. Figure 1 illustrates the specific electrode placements used for EEG, ECG, and EOG signal acquisition in this study. Figure 2 displays a photograph of a participant wearing the data collection equipment during the acquisition process.

This experiment has received approval from the Ethics Committee of ShanghaiTech University, with all participants having provided their written informed consent and being fully acquainted with the experimental procedures before the commencement of the study (Approval Number: ECSHU 2024-072).

### 2.2 Experimental procedure

In this study, multimodal data, including EEG, were collected from 10 participants during a task designed to induce attention levels. All participants were free from any neurological or psychiatric disorders. The experiments were conducted starting at 2:00 p.m. to ensure that all participants had sufficient time for a lunch break, thereby minimizing the impact of fatigue on attention level. Each participant's session lasted between one and two hours.

The experimental protocol was based on the AX-CPT paradigm (van der Linden et al., 2006). The AX-CPT paradigm involves presenting four-character groups sequentially, where each group is displayed one character at a time. Participants were required to view several groups of characters and make rapid judgments, while their reaction times and accuracy were recorded to induce an attentional state.

**Figure 3:**
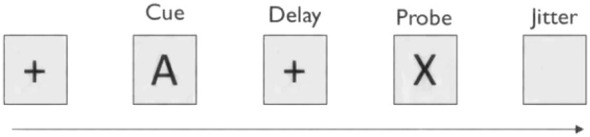
The order of character appearance in AX-CPT paradigm.

In each group of characters, the sequence consists of +, M, +, and N, where M and N represent arbitrary uppercase letters as shown in Figure 3 (with a high probability of being A and X). Each character is displayed at the center of the screen for several hundred milliseconds(average duration of 200 ms). Initially, a “+” is the fixation point, followed by an uppercase letter as a cue stimulus. Subsequently, “+” and a second uppercase letter appear in sequence. If the first letter is A and the second letter is X, participants must quickly respond with “1” after the second letter appears. If the first letter is A but the second letter is not X, the response should be “2.” If the first letter is not A, participants should respond with “2” immediately after the first letter disappears. Responses to the two types of judgments were executed via different keyboard keys, and the experiment required participants to make judgments both quickly and accurately. The duration of each “+” is several hundred milliseconds(average duration of 800 ms), followed by a rapid flashing of uppercase letters. To ensure effective attention level guidance, the duration of the “+” is not constant.

Previous studies have used reaction time in the AX-CPT paradigm as a metric for attention level and assigned different levels of attentional intensity based on the distribution of reaction times (Wan et al., 2021). However, in this experiment, the AX-CPT paradigm was employed solely to induce attention level in participants, without using reaction time as an index of attention level. Using real-time reaction time as an attention level measure could result in frequent alternations between attentive and inattentive states, which would complicate subsequent data processing and segmentation. Moreover, even in the absence of focused attention, the rapid changes in letters during the experiment could transiently enhance participants' attention level, thus shifting their EEG signals toward a more focused state. Given the variability of reaction times across individuals, relying on this measure might also lack reliability. Based on subjective feedback from participants, the AX-CPT paradigm effectively induced different levels of attentional states.

**Figure 4:**
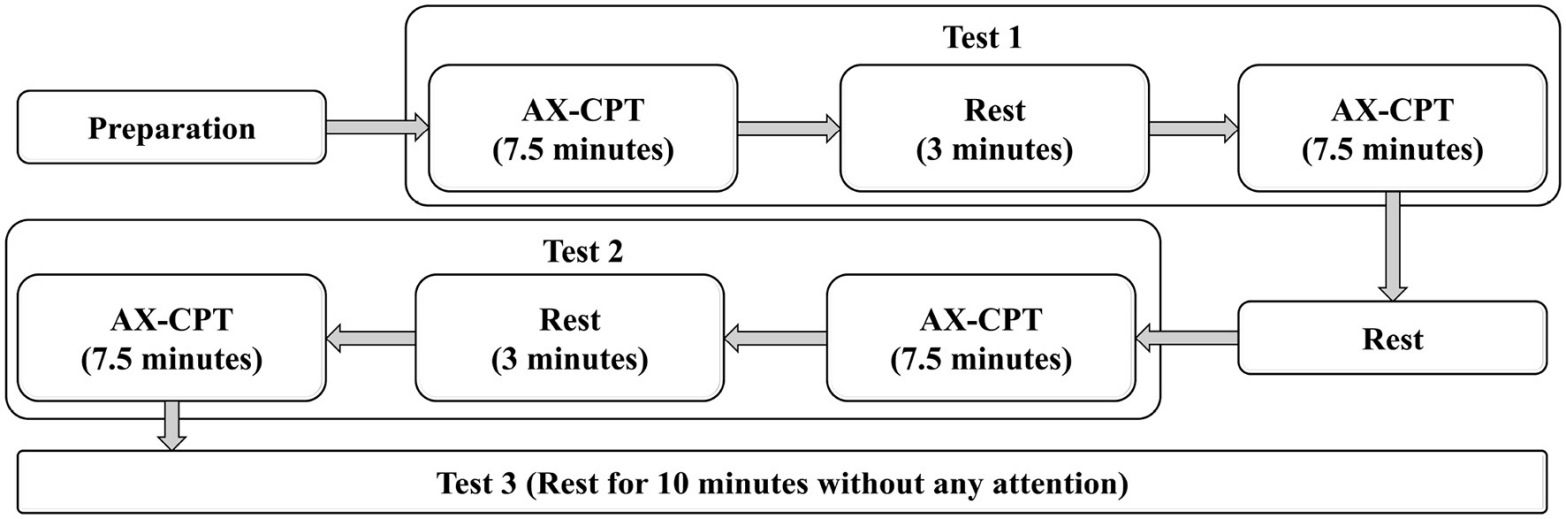
The complete process of multimodal data acquisition experiment.

The experiment was conducted in an electromagnetically shielded room, with each participant undergoing two continuous rounds of the procedure. Each round comprised two phases of the AX-CPT experiment, each consisting of 200 character sets, followed by a rest phase. To suppress alpha waves, participants were instructed to keep their eyes open during the rest phase. In the first round of the AX-CPT, participants were required to focus intently on the screen and respond as quickly as possible to the letters, which were classified as high attention level. In the second round of the AX-CPT, participants were asked to attend to the screen more casually and provide correct judgments within the duration of each character set, categorized as moderate attention level. The third round involved a rest period during which white noise designed to induce a relaxed state was played. During this time, participants were instructed not to engage in active thinking or attention, which was classified as low attention level. In the first two rounds of the experiment, participants were required to engage in a total of 15 min of attention tasks, while the rest phase in the third round lasted for 10 minutes. To ensure that participants followed the instructions as required, all participants received standardized task instructions and completed a practice session prior to the formal experiment. During data acquisition, clear requirements regarding reaction time and response accuracy were set for different experimental phases, and continuous supervision was provided throughout the session. Physiological signals were monitored in real time to verify task effectiveness. All participants included in the final analysis demonstrated good task compliance, with no obvious violations of the instructions observed. The complete experimental process is shown in sequence in Figure 4.

## 3 Data preprocessing

After data acquisition, a series of preprocessing steps were performed to ensure data quality and prepare for subsequent analysis. First, to reduce computational load while preserving the frequency components of interest, the raw data were downsampled from their original high sampling rate to 200 Hz. This process employed a Fourier transform-based frequency-domain resampling method with automatic application of anti-aliasing filtering to prevent high-frequency components from folding into the lower frequency bands. Subsequently, multi-stage filtering was applied: a notch filter was first used to eliminate power line interference, followed by a 2–50 Hz bandpass filter to remove low-frequency drift and high-frequency noise while retaining neural oscillations associated with attentional states. All filtering operations were implemented using zero-phase finite impulse response (FIR) filters to preserve the temporal relationships within the signals.

For each participant's data, segmentation was performed according to the experimental design. The multimodal physiological data for each participant were arranged in chronological order corresponding to high, medium, and low attention levels, with event markers precisely at the onset and offset of each attention level (markers were placed at the beginning of each condition). The three-minute rest periods of Test 1 and Test 2 were already removed during acquisition to ensure they did not interfere with the collected data. Based on these event markers, the continuous waveform data for each participant were segmented into three independent data segments representing different attention levels. To ensure classification reliability, these temporal segments were non-overlapping, and a portion of data was discarded from the beginning and end of each segment to achieve a duration of approximately 600 seconds per segment. This approach eliminated potential interference from transitional periods between different attentional states, ensuring that only relatively stable portions of each attentional state were retained for subsequent analysis.

**Figure 5:**
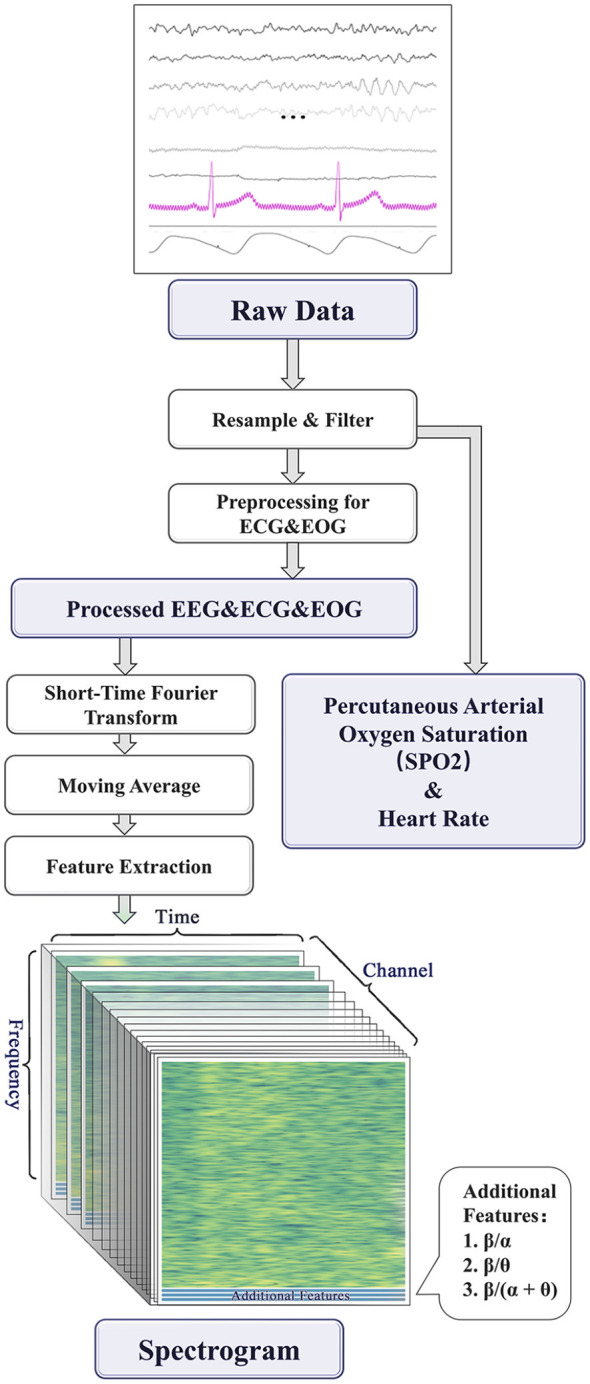
The overall processing workflow of the raw data.

The overall processing workflow of the raw data is illustrated in Figure 5.

According to standard preprocessing practices for ECG signals, baseline wandering was removed using morphological operations, specifically opening and closing operations applied in the time domain (Hoglinger, 2016). To achieve baseline wandering removal, the following operations were defined:

**Erosion Operation:**


$$\begin{array}{l} \left(f\Theta k\right)\left(n\right)=\underset{m=0...M-1}{\min}f\left(n+m\right)-k\left(m\right);\\ \;n=\left(0,1,...,N-M\right) \end{array}$$ (1)

**Dilation Operation:**


$$\begin{array}{l} \left(f⊕k\right)\left(n\right)=\underset{m=0...M-1}{\max}f\left(n-m\right)+k\left(m\right);\\ \;n=\left(M-1,M,...,N-1\right) \end{array}$$ (2)

**Opening Operation:**


$$\begin{array}{l} \left(f○k\right)\left(n\right)=\left(f\Theta k⊕k\right)\left(n\right) \end{array}$$ (3)

**Closing Operation:**


$$\begin{array}{l} \left(f\cdot k\right)\left(n\right)=\left(f⊕k\Theta k\right)\left(n\right) \end{array}$$ (4)

In the above formula, *f*(*n*) represents the original signal function with a total duration of *N*, and *k*(*m*) is the linear structuring element with a total duration of *M*, where both *N* and *M* denote the number of sampling points. Θ denotes the Erosion operator, ⊕ denotes the Dilation operator, ° represents the Opening operation, and · represents the Closing operation. By applying this operation, and selecting appropriate values for *M* and *k*(*m*), the baseline waveform can be directly obtained through the morphological operation. The specific values of *N* and *M* were determined by visually inspecting the ECG waveforms before and after processing in real time.


$$\begin{array}{l} f_{b}\left(n\right)=\left(\left(f○k\cdot k\right)/2+\left(f\cdot k○k\right)/2\right)\left(n\right) \end{array}$$ (5)

$$\begin{array}{l} f_{baseline}\left(n\right)=\left(\left(f_{b}○k\cdot k\right)/2+\left(f_{b}\cdot k○k\right)/2\right)\left(n\right) \end{array}$$ (6)

**Figure 6:**
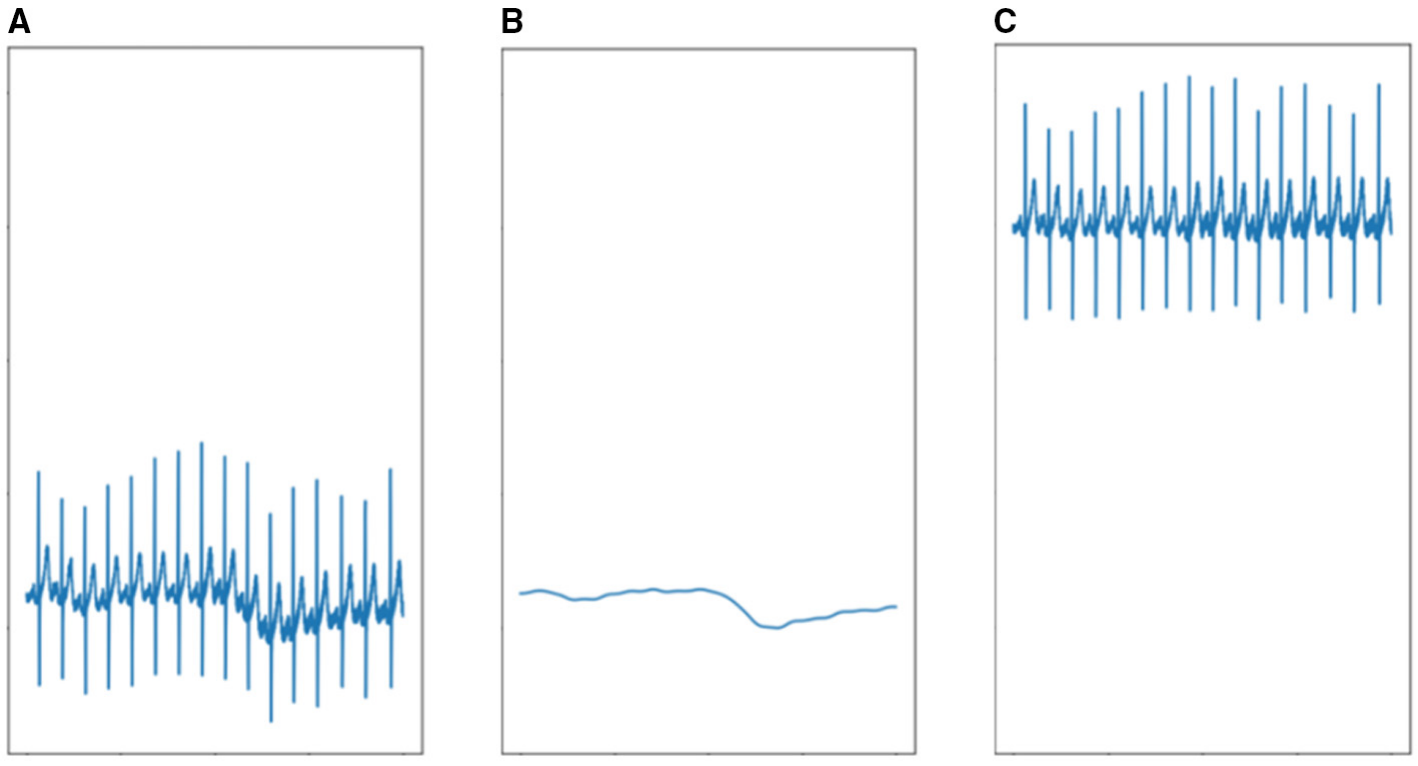
Qualitative illustration of ECG waveform before and after baseline wander removal (axes omitted for visual clarity). **(A)** The raw signal of ECG. **(B)** Baseline of ECG. **(C)** ECG after removing baseline wandering.

In this case, Figure 6A represents the original signal, and *f*_*baseline*_(*n*) represents the baseline shown in Figure 6B. By subtracting this baseline from the original signal, the processed signal illustrated in Figure 6C can be obtained.

For the electrooculography signals, the vertical EOG and horizontal EOG components can be obtained by computing the difference between the two signals that are perpendicular to each other.

We employed the Short-Time Fourier Transform (STFT) based on the Blackman window. A window length of 9 seconds with a step size of 1 second was used. The window length was chosen to provide sufficient frequency resolution while maintaining temporal resolution for tracking dynamic changes in physiological signals; specifically, 9 seconds captures multiple cardiac cycles and EEG oscillations for stable spectral estimation. The 1-second step size with high overlap (approximately 88.9%) ensures smooth temporal transitions. The Blackman window was selected for its superior side-lobe suppression to minimize spectral leakage.

For each windowed signal segment, the Fourier Transform was applied, and the logarithm of the signal power spectrum was computed. This process yielded power spectrograms for multiple modalities, including EEG (11 channels), EOG (2 channels), and ECG (1 channel). The resulting features are three-dimensional time-frequency representations with dimensions of [time frames × frequency bins × channels]. These spectrograms were subsequently converted into two-dimensional feature maps based on channels and frequencies, as described in the following section.

The formula for the Blackman window is as follows:


$$\begin{array}{l} w\left(\hat{t}\right)=0.42-0.5\cos\frac{2\pi \hat{t}}{Width-1}+0.08\cos\frac{4\pi \hat{t}}{Width-1},\\ \;0\leq \hat{t}<Width \end{array}$$ (7)

Here, *Width* denotes the width of the Blackman window.

By applying the Short-Time Fourier Transform to the waveform at each sampled time point, and moving the sampling window accordingly, the final time-frequency representation of frequencies below 30 Hz is derived from the original data. The time-frequency representation reflects the power distribution across different frequencies at each sampling time point. Subsequently, the data undergoes compression and denoising, such that each unit on the frequency scale represents a 0.33 Hz power distribution, and smoothing is applied to the power of each frequency unit on the time scale to mitigate noise effects.

Research indicates that different frequency bands of EEG activity can be associated with specific physiological states, and many studies consider the power ratios between specific frequency bands as crucial indicators of attention states. For instance, studies have shown that the power of EEG β-waves relative to α-waves and θ-waves is correlated with attention levels (Coelli et al., 2018). Therefore, for each channel, the features β/α, β/θ, and β/(α+θ) were calculated, where α, β, and θ represent the power of α-waves, β-waves, and θ-waves, respectively.

## 4 Model construction

After performing STFT on each channel of every modality, we obtained power spectrograms for multiple modalities, including EEG (11 channels), ECG (1 channel), EOG (2 channels), and pulse (1 channel). These data reflect the signal strength distribution at different time points. To integrate this information, we converted the spectrograms of each modality into two-dimensional feature maps based on channels and frequencies. These feature maps were then combined with the time-domain data from the remaining modalities to serve as input for the neural network.

**Figure 7:**
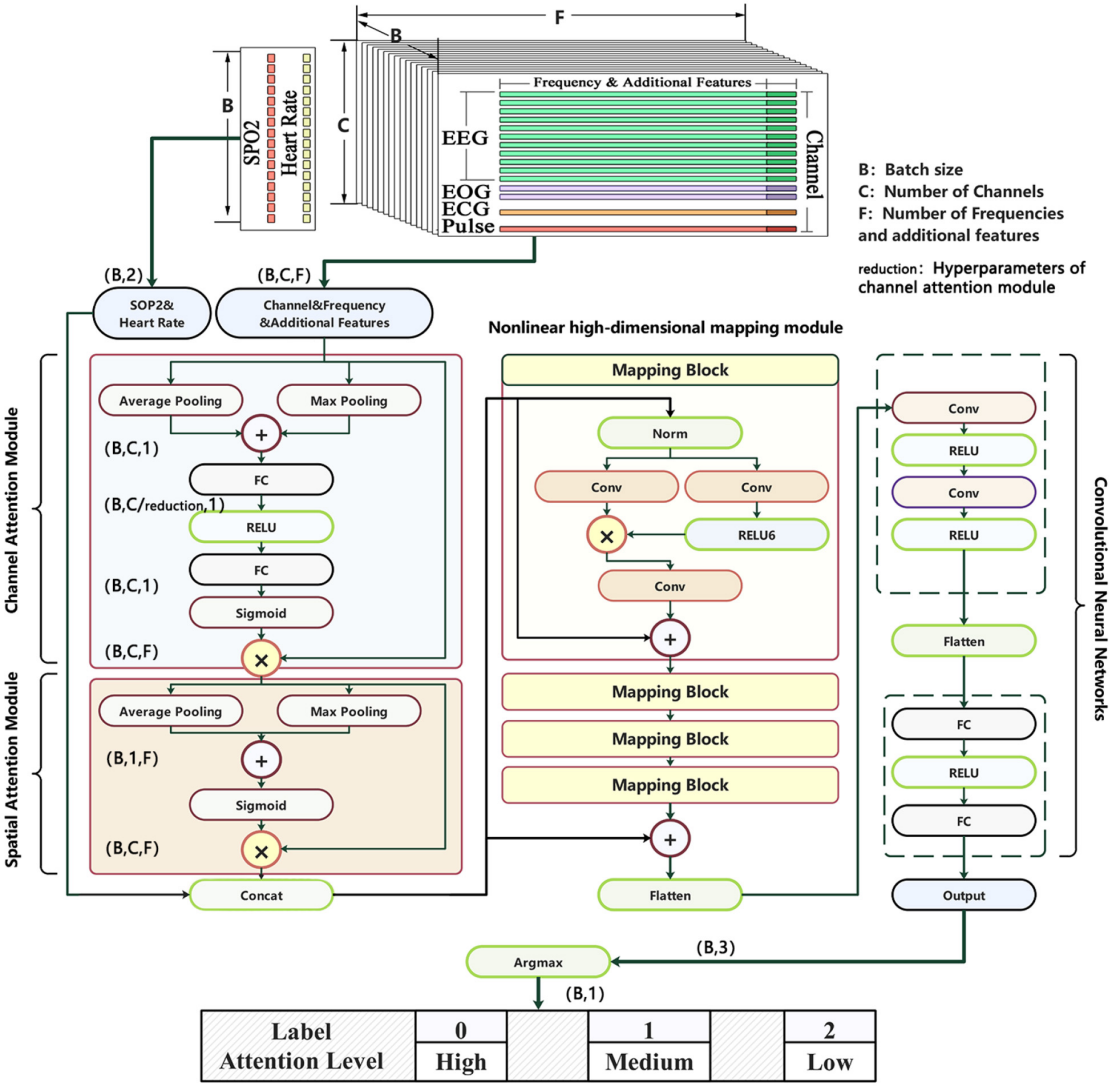
The overall structure of the Multifeature Enhancement Attention Network.

Overly complex models may lead to severe overfitting, affecting the practical performance of predictions. Thus, a model architecture that ensures both lightweight design and in-depth analysis of multimodal data features is necessary. Drawing on theories such as CBAM (Woo et al., 2018) and Star Operation (Ma et al., 2024), we developed a specialized model for multimodal brain-computer interfaces, named the Multifeature Enhancement Attention Network(MEAN). The CBAM introduces an Attention Module whose attention maps are inferred from channel and spatial dimensions. These attention maps are then multiplied with the feature maps for adaptive feature refinement. Furthermore, the study demonstrates that element-wise multiplication of tensors, known as the Star Operation, effectively integrates features from different subspaces, making it suitable for efficient and compact network architectures. MEAN is designed to fully leverage the characteristics of different modalities and deeply explore the rich information contained within them. The overall architecture of the model is illustrated in Figure 7.

### 4.1 Attention module

Channel attention operates along the sensor dimension, adaptively recalibrating the feature responses of different channels to achieve global optimization of information contributions from distinct brain regions. Spatial attention, in contrast, operates on the two-dimensional feature representation constructed from spectrograms (i.e., the channel-frequency plane), aiming to capture discriminative patterns within local frequency bands—specifically, the energy responses of particular channels at specific frequency ranges. In other words, channel attention performs cross-channel feature selection, while spatial attention focuses on locally salient regions within the joint channel-frequency representation. This cascaded attention architecture has been demonstrated in multiple studies to effectively extract complementary features and enhance model performance in physiological signal analysis tasks.

**Figure 8:**
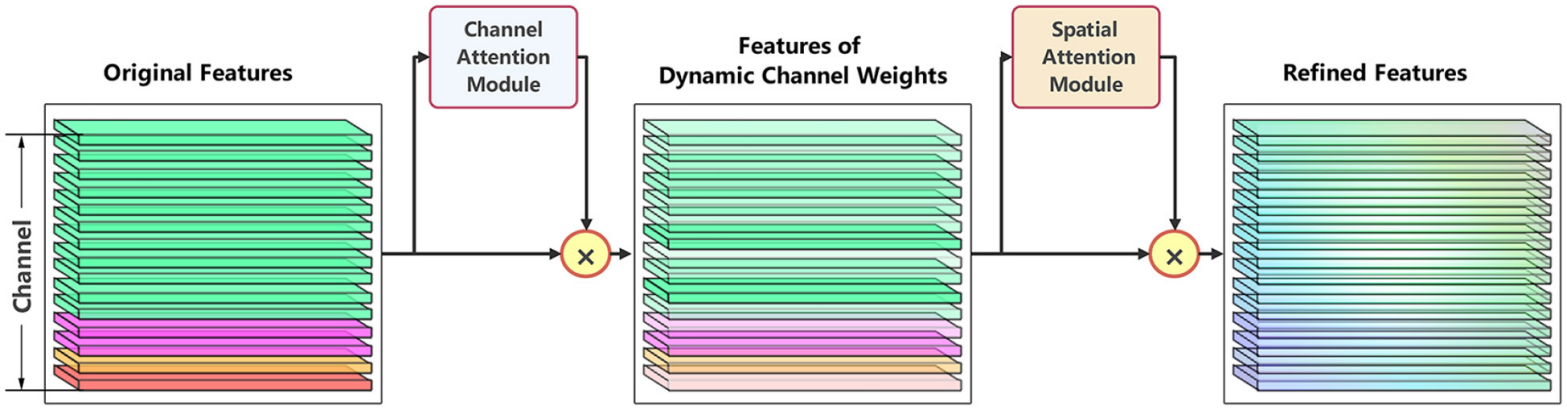
Schematic diagram of multimodal features passing through channels and spatial attention modules.

By incorporating both channel attention mechanisms and spatial attention mechanisms in series, the model explicitly models the dependencies between channels and the importance of features in the spatial domain. The channel attention module enables the network to adaptively learn the relative importance of each channel within the frequency features and assigns different weights to each channel, enhancing the model's ability to capture critical information from multimodal data. Spatial attention module, on the other hand, utilizes a weight matrix to highlight important spatial features while suppressing irrelevant spatial information. Figure 8 illustrates the process of original data passing through the attention modules.

The core steps of the channel attention module are Squeeze and Excitation (Hu et al., 2018). The Squeeze operation collects global information along the spatial dimensions and compresses it into a global vector representation. Here, the average of Average Pooling and Max Pooling is used as the method for extracting global information. The Excitation operation is responsible for performing a nonlinear transformation on the global vector and generating a weight vector using the Sigmoid function. This weight vector is then used to assign different dynamic weights to each modality, adaptively emphasizing useful modalities while suppressing less useful ones. The Excitation functionality is implemented using an FC-RELU-FC construction, which not only captures dependencies between channels but also enables parameterization of the gating mechanism.

The spatial attention module operates on principles similar to those of the channel attention module. The processed frequency domain data highlights relatively important power information and features from two dimensions, compared to the original data. These are then concatenated with two time-domain modality indicators (SPO2 and Heart Rate) to form a more effective feature representation.

### 4.2 Nonlinear high-dimensional mapping module

**Figure 9:**
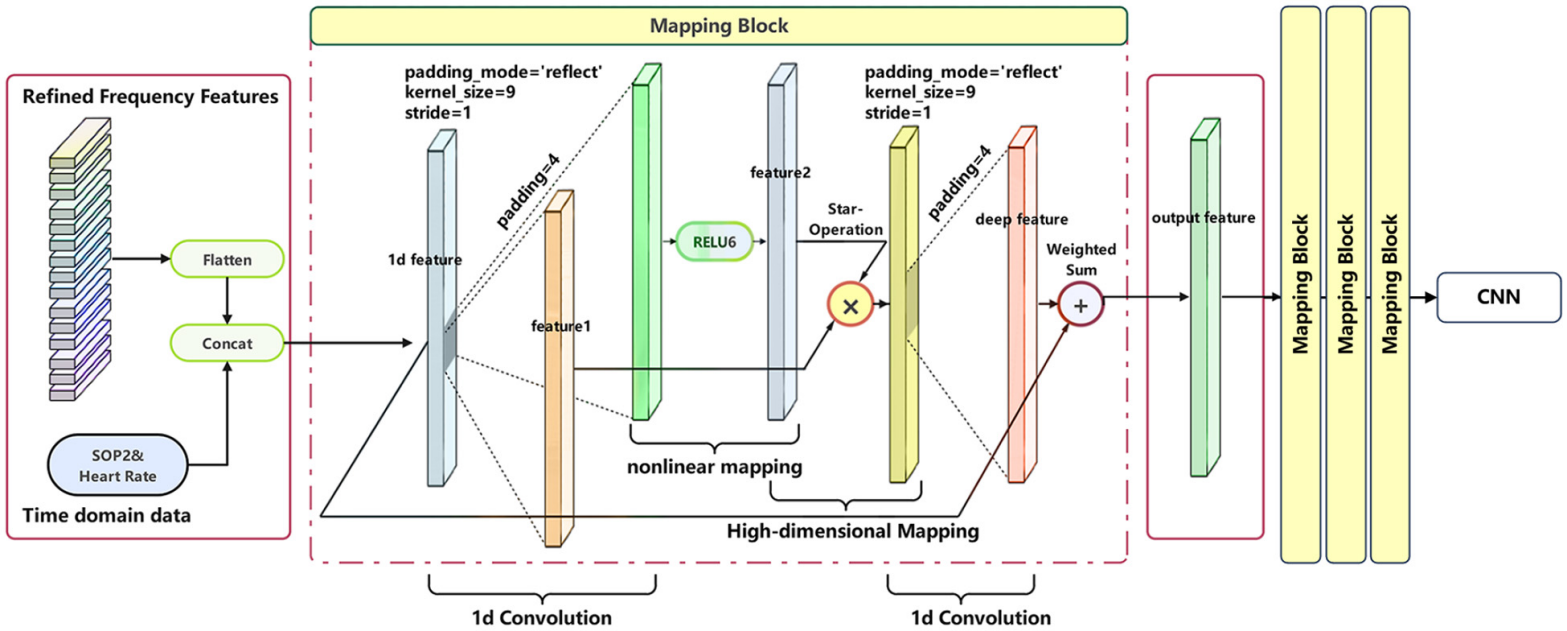
Schematic diagram of refined multimodal features passing through the mapping block module.

The Star Operation is a method that integrates features from different subspace representations through element-wise multiplication. This technique demonstrates excellent performance and efficiency across various research fields, including NLP and computer vision (Liu et al., 2025; Lu et al., 2025), where it has been successfully applied. In the Nonlinear High-dimensional Mapping Module, the model employs four identical Mappin g Blocks. Each Mapping Block utilizes a convolutional layer followed by a convolutional layer combined with the RELU6 function to extract two sets of shallow features. These features are then fused through element-wise multiplication (i.e., Star Operation), mapping them into a higher-dimensional nonlinear feature space. Figure 9 illustrates the process of further feature extraction for multimodal physiological signals within each Mapping Block following the channel attention module. First, multi-channel signals are flattened into one-dimensional vectors and concatenated with two types of temporal data–SPO2 and Heart Rate–to form a one-dimensional tensor. Subsequently, this tensor is passed through a linear mapping block to obtain the feature representation denoted as feature 1 in the diagram, and through a nonlinear mapping block to obtain feature 2. The linear mapping is implemented via one-dimensional convolution, while nonlinearity is introduced through the activation function RELU6. Finally, the Star operation is applied to feature 1 and feature 2 to derive implicit high-dimensional features, which are then combined with the original one-dimensional tensor via weighted summation to integrate characteristics from both representations. Compared to traditional summation or widening the network, this mapping approach employs cross-channel feature multiplication, akin to polynomial kernel functions, enabling nonlinear high-dimensional mapping. The function of all mapping blocks is to project the feature tensor into a higher-dimensional subspace, thereby further increasing its implicit dimensionality. The choice of using four mapping blocks is intended to balance the model's mapping capability with its lightweight design.

In a single layer of a neural network, the star operation is commonly denoted as $\left(\begin{array}{c} {\text{W}}_{1}^{\text{T}}\text{X}+{\text{B}}_{1} \end{array}\right)*\left(\begin{array}{c} {\text{W}}_{2}^{\text{T}}\text{X}+{\text{B}}_{2} \end{array}\right)$, which represents the element-wise fusion of features from two linear transformations. For brevity, the weight matrix and bias can be combined and denoted as $\text{W}=\left[\begin{array}{c} \text{W}\\ \text{B} \end{array}\right]$, with a similar notation $\text{X}=\left[\begin{array}{c} \text{X}\\ 1 \end{array}\right]$, thereby simplifying the overall expression to $\left(\begin{array}{c} {\text{W}}_{1}^{\text{T}}\text{X} \end{array}\right)*\left(\begin{array}{c} {\text{W}}_{2}^{\text{T}}\text{X} \end{array}\right)$. For an individual element, this can be represented by the following process:


$$\begin{array}{l} w_{1}^{\text{T}}x*w_{2}^{\text{T}}x=\left(\overset{d+1}{\underset{i=1}{\sum }}w_{1}^{i}x^{i}\right)*\left(\overset{d+1}{\underset{j=1}{\sum }}w_{2}^{j}x^{j}\right)\\ \;=\overset{d+1}{\underset{i=1}{\sum }}\overset{d+1}{\underset{j=1}{\sum }}w_{1}^{i}w_{2}^{j}x^{i}x^{j}\\ \;=\alpha_{1}^{1}x^{1}+\cdots +\alpha_{\left(2,3\right)}x^{2}x^{3}+\cdots +\alpha_{\left(d+1,d+1\right)}x^{d+1}x^{d+1} \end{array}$$ (8)

Where:


$$\begin{array}{l} \alpha_{\left(i,j\right)}=\{\begin{array}{cc} w_{1}^{i}w_{2}^{j} & \;\text{if}\;i=j,\\ w_{1}^{i}w_{2}^{j}+w_{1}^{j}w_{2}^{i} & \;\text{if}\;i\neq j. \end{array} \end{array}$$ (9)

Finally, we obtain a polynomial with $\frac{\left(d+2\right)\left(d+1\right)}{2}$ terms, where each term, except those containing *x*^*d*+1^(i.e., 1), exhibits a nonlinear dependence on *x*, indicating that they are independent and implicit dimensions. Thus, the star operation, performed in a *d*-dimensional space, achieves representation in feature space with an implicit dimensionality of approximately $\frac{d^{2}}{2}$, significantly expanding the feature dimensions without substantially increasing the model size. Clearly, with multiple layers of stacked star operations, the size of the implicit dimensions can grow almost exponentially.

After processing through the four Mapping Blocks, the original features are transformed into latent features that potentially contain highly complex and abstract information. Subsequently, these latent features are combined with the original features through weighted summation, with the weights automatically adjusted during the model's training process through gradient descent. This mechanism not only enables the model to automatically focus on and enhance relevant information but also improves the model's generalization ability and classification accuracy.

In the Mapping Block of MEAN, convolutional layers are employed instead of fully connected layers to process features. This choice is intended to reduce the number of model parameters, thereby enhancing the model's operational efficiency and generalization capability.

Finally, the integrated feature representations are fed into a Convolutional Neural Network for the final three-class classification task.

## 5 Results

The model training and testing are conducted using five-fold cross-validation. The dataset is divided into training and testing sets based on temporal relationships. For each attention level, the preprocessed data are divided into five equal parts along the time axis. One part is selected as the test set, and the remaining four parts are used as the training set. This temporal division prevents overlap between training and testing sets, which could otherwise lead to inflated prediction accuracy. To assess the model's stability, a fixed proportion of data is randomly sampled from both the training and test sets for each iteration. This process is repeated five times, resulting in a total of 50 training and testing cycles throughout the entire procedure. The detailed data dimensionality and feature composition for each subject under this 5-fold cross-validation framework are presented in Table 1, where consistent feature computation is applied across all frequency-domain modalities (EEG, ECG, EOG, Pulse) to ensure structural uniformity.

**Table 1:** Summary of dataset dimensionality and feature composition for each subject: Total instances from 5-fold cross-validation with consistent feature structure across all frequency-domain modalities, resulting in a uniform 15 × 93 feature matrix per instance.

Subject	Number of instances per class			Total instances	Feature dimensions		Total features
	High	Medium	Low		Frequency domain	Time domain	
S1	3,809	3,943	3,393	11,145	(15,93)	2	1,397
S2	3,893	4,269	3,943	12,105			
S3	3,929	4,035	3,936	11,900			
S4	6,736	6,345	4,189	17,270			
S5	4,046	3,889	3,905	11,840			
S6	4,747	4,782	4,176	13,705			
S7	5,568	5,590	3,857	15,015			
S8	5,400	5,696	3,759	14,855			
S9	5,046	4,412	4,742	14,200			
S10	4,258	4,273	4,179	12,710			
**Frequency domain feature composition:**							
• Total channels: 15 (EEG: 11 channels, ECG: 1 channel, EOG: 2 channels, Pulse: 1 channel)							
• Each channel: 93 features (90 frequency features + 3 additional EEG-derived features)							
• Total frequency features: 15 channels × 93 features = **1395 features**							
**Time domain feature composition:**							
• SPO2 and Heart Rate: 2 features							
**Total feature dimension per instance: 1395 (frequency) + 2 (time)** **=** **1397 features**							

All 93 features—including three originally derived from EEG characteristics—were generated for every channel (EEG, ECG, EOG, and pulse) to maintain structural consistency across modalities. For non-EEG channels, these three features serve as dimensional placeholders to ensure a uniform input tensor shape for subsequent model processing.

For a comprehensive performance evaluation, we compare our model with several existing models. The convolutional neural network used as the first comparison model has exactly the same structure and parameters as the convolutional neural network that follows the four Mapping Blocks in the proposed Multifeature Enhancement Attention Network, which serves both as a performance benchmark and an ablation study to validate the effects of individual modules. The second model employs a Recurrent Neural Network with 128 units in the hidden layers and two stacked layers, culminating in a linear fully connected layer for classification. The third model is a Long Short-Term Memory network, a variant of RNNs that incorporates gating mechanisms (input gate, forget gate, and output gate) to manage and control the information flow within the units. This LSTM model has the same number of hidden units and layers as the RNN model. The fourth model is a Support Vector Machine with a linear kernel, widely used in attention level prediction research. To ensure a fair comparison, we now explicitly state that all classifiers in the comparative study–including the first CNN model and other baseline models–were provided with multimodal input under identical conditions. Specifically, the preprocessing steps for each modality remained consistent with the methods described earlier in the paper. Before being fed into any classifier, features from all modalities were concatenated and flattened into a one-dimensional tensor. This standardized input pipeline guarantees that every model received the same fused multimodal representation, allowing the comparison to focus solely on architectural differences. All baseline models described above were implemented from scratch using PyTorch, based on the methodological descriptions in their respective foundational literature.

Table 2 shows the average accuracy of different models across subjects, the standard deviation of the five-fold cross-validation accuracy for each model and subject, and the p-values obtained from paired t-tests comparing the performance of other models with that of the model in this study. All *p*-values are less than 0.05, indicating statistically significant differences between the other models and MEAN, suggesting that the differences among the models are highly reliable.

**Table 2:** Accuracy, standard deviation, and *p*-value of different models and subjects.

Acc(%)	Subject	Model				
		CNN	RNN	LSTM	SVM	MEAN
	S1	91.41	92.55	89.58	91.00	96.68
	S2	87.56	91.96	92.45	93.17	96.86
	S3	87.26	91.80	93.71	93.85	97.83
	S4	98.73	80.57	97.42	99.66	99.68
	S5	94.82	94.39	92.83	94.20	98.80
	S6	94.01	84.88	87.06	96.17	96.95
	S7	88.99	84.15	86.88	91.84	92.39
	S8	96.53	90.70	90.19	96.59	97.61
	S9	84.61	82.02	82.60	86.41	90.79
	S10	79.30	73.50	73.81	82.18	84.83
	Average	90.32	86.65	88.65	92.51	95.24
Std(%)	Subject	Model				
		CNN	RNN	LSTM	SVM	MEAN
	S1	9.52	4.70	9.51	12.89	1.93
	S2	13.53	9.09	8.45	8.36	4.81
	S3	11.18	6.43	5.10	4.49	2.67
	S4	1.23	10.17	0.92	0.56	0.24
	S5	1.48	2.61	1.63	3.42	0.56
	S6	3.91	8.59	8.64	2.07	1.41
	S7	10.48	7.08	8.14	7.36	10.62
	S8	3.39	5.37	6.67	3.26	2.59
	S9	10.14	9.11	10.32	8.56	9.18
	S10	10.39	14.49	15.52	11.36	9.28
	Average	7.52	7.76	7.49	6.23	4.33
P-value		Model				
		CNN	RNN	LSTM	SVM	MEAN
		2.19e-06	7.65e-11	5.75e-10	3.48e-2	/

MEAN achieves the best average predictive performance, followed by SVM. The performance of CNN also indicates that the Attention Module and Nonlinear High-dimensional Mapping Module significantly enhance the model's effectiveness.

To further demonstrate that the introduction of additional modalities beyond EEG can indeed enhance predictive performance, we conducted a modality ablation experiment to examine the prediction accuracy when using only specific modalities. SVM was specifically chosen for this ablation analysis due to its well-established stability and interpretability in physiological signal classification. As a non-deep learning baseline with widely recognized reliability, SVM provides a more robust benchmark for assessing the individual contribution of each modality. As shown in Table 3, despite the variation in the number of channels across different modalities, which may correlate prediction accuracy with the number of channels, the ECG and EOG signals alone still exhibited performance significantly surpassing the 33% random baseline. This indicates that the inclusion of these two modalities contributes substantially to the prediction task. In contrast, although the heart rate, SPO2, and pulse modalities led to a slight improvement in overall prediction accuracy for most subjects, the presence of significant noise interference in these signals (e.g., in subjects S2, S5, and S7) caused a notable decline in prediction performance. Therefore, their importance is considered to be lower than that of the three primary modalities.

**Table 3:** SVM accuracy under different modalities.

Subject	SVM accuracy(%)					
	EEG 11 channel	EOG (2 channel)	ECG (1 channel)	EEG+EOG	EEG+EOG+ECG	All modalities
S1	76.62	69.88	45.32	84.58	90.21	91.00
S2	86.21	76.48	74.87	86.24	94.59	93.17
S3	83.51	55.12	93.26	86.22	89.45	93.85
S4	95.89	93.10	76.68	98.91	99.17	99.66
S5	95.58	69.79	79.65	96.36	97.26	94.20
S6	83.11	84.02	70.97	91.24	93.89	96.17
S7	78.50	76.82	77.05	86.88	92.21	91.84
S8	89.75	97.06	91.76	94.88	95.50	96.59
S9	84.53	70.46	58.02	85.62	85.90	86.41
S10	75.51	62.70	71.75	76.43	82.08	82.18
Average	84.92	75.54	73.93	88.74	92.03	92.51

The average accuracy achieved is comparable to the results from previous studies, and the inclusion of additional modalities such as ECG and EOG has significantly enhanced the predictive accuracy. Although SVM performs exceptionally well with smaller datasets, it does not perform as effectively with larger datasets. Given MEAN's potential in analyzing higher-dimensional latent features, the model has demonstrated superior performance in the project. As the dataset expands, MEAN is expected to further improve stability and prediction accuracy.

To more comprehensively evaluate the overall performance of the model, we derive the True Positives (TP), False Positives (FP), True Negatives (TN), and False Negatives (FN) for each focus state of each subject from the confusion matrix. We then compute the corresponding Precision, Recall, and F1-score. Specifically, for any given class *k*, the formulas for these metrics are as follows:


$$\begin{array}{l} TP_{k}=\overset{N}{\underset{i=1}{\sum }}\delta \left(y_{i}=k\right)\cdot \delta \left({\hat{y}}_{i}=k\right) \end{array}$$ (10)

$$\begin{array}{l} FP_{k}=\overset{N}{\underset{i=1}{\sum }}\delta \left(y_{i}\neq k\right)\cdot \delta \left({\hat{y}}_{i}=k\right) \end{array}$$ (11)

$$\begin{array}{l} TN_{k}=\overset{N}{\underset{i=1}{\sum }}\delta \left(y_{i}\neq k\right)\cdot \delta \left({\hat{y}}_{i}\neq k\right) \end{array}$$ (12)

$$\begin{array}{l} FN_{k}=\overset{N}{\underset{i=1}{\sum }}\delta \left(y_{i}=k\right)\cdot \delta \left({\hat{y}}_{i}\neq k\right) \end{array}$$ (13)

where δ(·) is the indicator function,*y*_*i*_ is the true label, and ŷ_*i*_ is the predicted label. The expression for the indicator function in the context of classification problems is as follows:


$$\begin{array}{l} \delta \left(condition\right)=\{\begin{array}{cc} 1 & \text{if }condition\text{ is true}\\ 0 & \text{if }condition\text{ is false} \end{array} \end{array}$$ (14)

Based on the above definitions, the formulas for Precision, Recall, and F1-score for each class can be further detailed:


$$\begin{array}{l} {\text{Precision}}_{k}=\frac{TP_{k}}{TP_{k}+FP_{k}} \end{array}$$ (15)

$$\begin{array}{l} {\text{Recall}}_{k}=\frac{TP_{k}}{TP_{k}+FN_{k}} \end{array}$$ (16)

$$\begin{array}{l} {\text{F1}}_{k}=2\cdot \frac{{\text{Precision}}_{k}\cdot {\text{Recall}}_{k}}{{\text{Precision}}_{k}+{\text{Recall}}_{k}} \end{array}$$ (17)

Accordingly, the comprehensive performance metrics obtained by MEAN are summarized in Table 4.

**Table 4:** Model evaluation metrics for each attention state category within each subject using MEAN.

Subject	Attention level	TP	TN	FP	FN	Precision	Recall	F1
S1	High	3,768	7,219	117	41	0.9699	0.9892	0.9795
	Medium	3,693	7,092	110	250	0.9711	0.9366	0.9535
	Low	3,314	7,609	143	79	0.9586	0.9767	0.9676
S2	High	3,776	8,161	51	117	0.9867	0.9699	0.9782
	Medium	4,209	7,522	314	60	0.9306	0.9859	0.9575
	Low	3,739	8,146	16	204	0.9957	0.9483	0.9714
S3	High	3,824	7,904	67	105	0.9828	0.9733	0.9780
	Medium	3,941	7,706	159	94	0.9612	0.9767	0.9689
	Low	3,877	7,932	32	59	0.9918	0.9850	0.9884
S4	High	6,725	10,522	12	11	0.9982	0.9984	0.9983
	Medium	6,323	10,892	33	22	0.9948	0.9965	0.9957
	Low	4,167	13,071	10	22	0.9976	0.9947	0.9962
S5	High	3,989	7,742	52	57	0.9871	0.9859	0.9865
	Medium	3,849	7,903	48	40	0.9877	0.9897	0.9887
	Low	3,860	7,893	42	45	0.9892	0.9885	0.9889
S6	High	4,602	8,817	141	145	0.9703	0.9695	0.9699
	Medium	4,644	8,739	184	138	0.9619	0.9711	0.9665
	Low	4,041	9,436	93	135	0.9775	0.9677	0.9726
S7	High	4,558	9,346	101	1,010	0.9783	0.8186	0.8914
	Medium	5,460	9,161	264	130	0.9539	0.9767	0.9652
	Low	3,853	10,379	779	4	0.8318	0.9990	0.9078
S8	High	5,321	9,393	62	79	0.9885	0.9854	0.9869
	Medium	5,462	9,048	111	234	0.9801	0.9589	0.9694
	Low	3,718	10,915	181	41	0.9536	0.9891	0.9710
S9	High	4,825	8,876	278	221	0.9455	0.9562	0.9508
	Medium	4,125	9,038	750	287	0.8462	0.9350	0.8883
	Low	3,941	9,177	281	801	0.9334	0.8311	0.8793
S10	High	3,577	7,395	1,057	681	0.7719	0.8401	0.8045
	Medium	3,260	7,758	679	1,013	0.8276	0.7629	0.7940
	Low	3,944	8,338	193	235	0.9533	0.9438	0.9485

**Figure 10:**
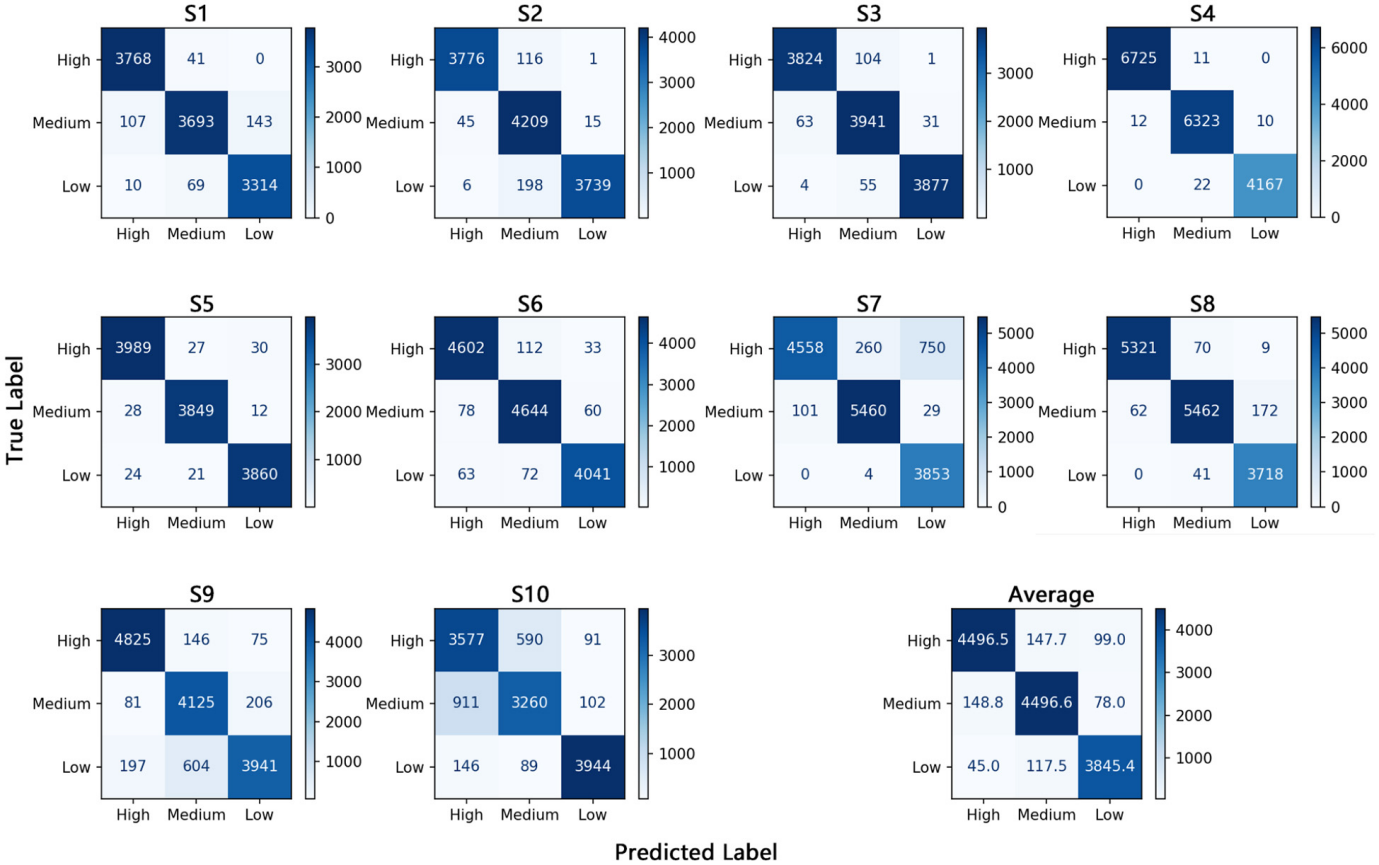
Confusion matrices for each subject and the average.

Furthermore, we can visualize the confusion matrices for each subject and the average confusion matrix based on the aforementioned data, providing a more intuitive representation of the model's classification performance, as shown in Figure 10.

Each subject's confusion matrix represents the aggregation of prediction results obtained from five-fold cross-validation, where each fold is repeated five times, resulting in a total of 25 predictions.

From the confusion matrix, it is evident that the model demonstrates excellent performance in classifying instances with low attention levels, while classification errors are relatively higher for instances with high and medium attention levels. In most cases, when the real attention level is high, if misclassified as either medium or low attention level, it is more frequently misclassified as medium attention level and rarely as low attention level. For instances with medium attention level, misclassifications into high and low attention levels occur with roughly equal frequency. For instances with low attention level, misclassifications into medium attention level are more common than those into high attention level. These results indicate that as attention level transitions from high to low, the features embedded in multimodal signals, primarily derived from EEG, also exhibit a continuous variation.

## 6 Discussion

The use of EEG to identify the level of human concentration has important practical significance. Real-time concentration monitoring has a very broad practical application prospect in fields such as mental health and cognitive training, autonomous driving and intelligent transportation, education, etc. With the assistance of other modalities such as electrooculography, the reliability of monitoring results can be further improved, providing further guarantees for its practical application.

In this study, we conducted a comprehensive evaluation of several classification models for attention-level analysis using EEG combined with multimodal signals. The models included Convolutional Neural Networks, Recurrent Neural Networks, Long Short-Term Memory networks, Support Vector Machines, and the proposed deep learning model MEAN. We employed five-fold cross-validation and performed temporal segmentation of the data to ensure the temporal independence between the training and testing sets. This approach allowed us to avoid temporal overlap between the training and testing sets, providing a more realistic assessment of the model's predictive capability.

Attention level classification is often binary, with three-class classification frequently including “drowsiness”. While drowsiness or sleepiness is an intuitive indicator of low attention level, the significant increase in alpha wave power during this state allows for relatively straightforward classification, thereby improving prediction accuracy. In EEG-based attention level prediction research, the lack of uniform classification standards and rigor makes it challenging to compare classification performance across different studies. We employed a three-class classification scheme (high attention level, moderate attention level, and low attention level) with an experimental procedure that gradually relaxed over time while requiring subjects to keep their eyes open to avoid increased alpha wave power. Consequently, accurate classification in this context presents a higher difficulty compared to other studies, therefore being of greater reference value.

Table 5 presents a chronological overview of recent research on EEG-based attention level prediction. As discussed in the literature, direct performance comparisons between studies are inherently limited due to substantial differences in datasets, experimental protocols, and classification schemes. Therefore, this table is positioned in the Discussion section not for rigorous benchmarking, but rather to provide readers with a comprehensive synthesis of the field's evolution and methodological diversity.

**Table 5:** Recent research on the prediction of concentration using EEG signals.

Reference	Year	Method	Classification task	Classes	Accuracy
Acı et al. (2019)	2019	EEG, SVM	mental attention state (3 classes)	focused, unfocused, drowsy	91.72%
Zennifa and Iramina (2019)	2019	EEG&ECG&NIRS, CFS+KNN	attention level (2 classes)	high attention, low attention	82.31%
Jeong and Jeong (2020)	2020	In-Ear EEG, ESN	attention state (2 classes)	attentive state, resting state	82.44%
Zhang et al. (2021)	2021	EEG, 3DCNN	attentive mental state (2 classes)	task period, rest period	81%
Toa et al. (2021)	2021	EEG, CAMNN	attention level (2 classes)	attentive behavior, inattentive behavior	92%
Nguyen et al. (2022)	2022	EEG, SVM	concentration state (2 classes)	concentration, rest	85%
Al-Nafjan and Aldayel (2022)	2022	EEG, Random Forest	attention level (2 classes)	focused state, negative state	96%
Salankar et al. (2023)	2023	EEG, MLP	cognitive attention (2 classes)	attentive, non-attentive	81%
Wu et al. (2023)	2023	EEG, LSTM-CNN	mental fatigue (2 classes)	positive, negative	TPR=97.02%
Xu X. et al. (2023)	2023	EEG, SVM	attention level (4 classes)	high, medium, low, non-externally directed	92%
Gupta et al. (2024)	2024	EEG, SVM	cognitive state (2 classes)	attentiveness, inattentiveness	91.68%
Hu F. et al. (2024)	2024	EEG, STFN-BRPS	driving fatigue (3 classes)	no/low drowsiness, moderate drowsiness, high drowsiness	92.43%
Peng et al. (2024)	2024	EEG, 3D-STCNN	driving fatigue (3 classes)	low load, medium load, high load	87.55%
Lee et al. (2024)	2024	EEG, CNN+LSTM	fatigue level (3 classes)	normal state, low fatigue, high fatigue	88.01%
This study	2024	EEG&ECG&EOG, MEAN	attention level (3 classes)	high attention level, moderate attention level, low attention level	95.24%

With this context in mind, it is worth noting that our proposed MEAN model achieves an accuracy of 95.24% on a three-class attention classification task. This result is particularly noteworthy given that multi-class classification (three or more levels) is generally more challenging than binary classification, which is employed by most of the higher-performing studies in the table. Thus, while direct comparisons are not appropriate, the performance of our model appears promising within the broader research context.

In this experiment, all deep learning models employed weight decay as a regularization technique to stabilize predictive performance, and Adam was used as the optimizer. Adam optimizes performance by computing adaptive learning rates for each parameter, which effectively manages updates across different parameters. This means each parameter has its unique learning rate, which helps address sparse gradients and gradients of varying scales during training. By combining momentum methods with adaptive learning rate techniques, the Adam optimizer leverages the advantages of both: it accelerates convergence and reduces oscillations during optimization, while also handling gradients with different scales and sparsity, thereby improving overall optimization stability.

**Figure 11:**
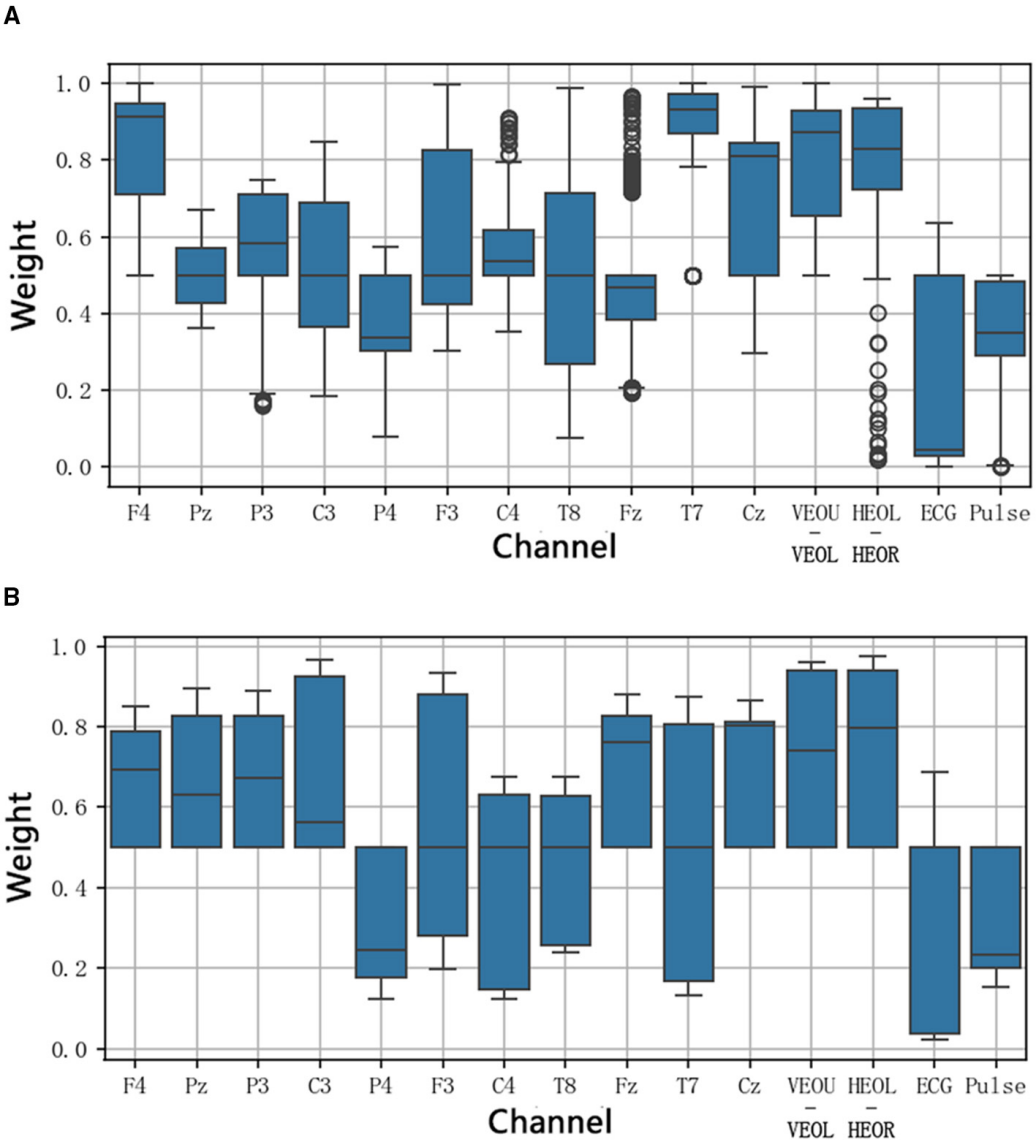
The boxplots of channel weights for the data from Fold1 to Fold5 of S4 and Fold3 to Fold5 of S8. **(A)** Fold1 to Fold5 of S4. **(B)** Fold3 to Fold5 of S8.

To further analyze the contribution of different channels to focus prediction, we selected Fold1 to Fold5 from S4 and Fold3 to Fold5 from S8, and extracted the channel weights from the channel attention module for these data sets and plotted them in the boxplot shown in Figure 11. From left to right, the channels include EEG channels F4, Pz, P3, C3, P4, F3, C4, T8, Fz, T7, Cz; EOG channels (vertical EOG: VEOU-VEOL, horizontal EOG: HEOL-HEOR); ECG; and Pulse.

Although there is variation in the channel weights among different subjects (potentially due to differences in electrode resistance during different experiments, which affects the quality of the signals collected), some common patterns can still be discerned. EEG channels exhibit considerable weight variability, but overall, EEG channels demonstrate higher weights in focus prediction. EOG channels also consistently perform strongly, indicating their significant role in focus prediction. In contrast, ECG and Pulse contribute relatively less.

## 7 Conclusion

This study provides a comprehensive evaluation of using multimodal data, including EEG, ECG, and EOG, to measure human attention levels and introduces the Multifeature Enhancement Attention Network model architecture. MEAN employs various attention mechanisms to dynamically and adaptively filter frequency and channel features, achieving superior feature extraction with a relatively simple architecture. We have demonstrated that the proposed MEAN model achieves an average accuracy of 0.9524, outperforming traditional deep learning models.

Furthermore, this study leverages the data fusion advantages of multimodal physiological signals by combining EEG with ECG and EOG, overcoming the limitations of single-modal signals in attention level prediction. EEG signals effectively reflect brain electrical activity and correlate strongly with attention states across different frequency bands. However, EEG signals are susceptible to noise and provide relatively narrow information. Including ECG and EOG signals as supplementary data significantly enhances the model's predictive accuracy. ECG signals provide physiological data related to heart rate variability, while EOG signals capture eye movement information. These additional physiological parameters offer a more comprehensive feature representation, improving the robustness and accuracy of attention level prediction.

The practical results confirm that integrating ECG and EOG multimodal data with EEG can significantly enhance attention level prediction performance. The design of the MEAN model substantially improves the handling of multimodal data. Through channel and spatial attention mechanisms, the model adaptively emphasizes useful information and suppresses redundant or noisy signals, which is crucial for processing data from different modalities. Particularly with limited sample sizes, MEAN's lightweight design and effective feature fusion help mitigate overfitting issues, resulting in higher prediction accuracy. Therefore, this study not only demonstrates the potential of MEAN in practical applications but also provides a reference for research on attention level prediction based on multimodal data.

## Data Availability

The raw data supporting the conclusions of this article will be made available by the authors, without undue reservation.

## References

[B1] Acı Ç. İ Kaya M. Mishchenko Y. (2019). Distinguishing mental attention states of humans via an eeg-based passive bci using machine learning methods. Expert Syst. Appl. 134, 153–166. doi: 10.1016/j.eswa.2019.05.057

[B2] Alirezaei M. Sardouie S. H. (2017). “Detection of human attention using EEG signals,” in 2017 24th National and 2nd International Iranian Conference on biomedical engineering (ICBME) (Tehran: IEEE), 1–5.

[B3] Al-Nafjan A. Aldayel M. (2022). Predict students' attention in online learning using eeg data. Sustainability 14:6553. doi: 10.3390/su14116553

[B4] Belle A. Hargraves R. H. Najarian K. (2012). An automated optimal engagement and attention detection system using electrocardiogram. Comput. Mathem. Methods Med. 2012:528781. doi: 10.1155/2012/528781 22924060 PMC3424596

[B5] Codispoti M. Mazzetti M. Bradley M. M. (2009). Unmasking emotion: Exposure duration and emotional engagement. Psychophysiology 46, 731–738. doi: 10.1111/j.1469-8986.2009.00804.x 19386053

[B6] Coelli S. Barbieri R. Reni G. Zucca C. Bianchi A. M. (2018). Eeg indices correlate with sustained attention performance in patients affected by diffuse axonal injury. Med. Biol. Eng. Comp. 56, 991–1001. doi: 10.1007/s11517-017-1744-5 29124529

[B7] Djamal E. C. Pangestu D. P. Dewi D. A. (2016). “EEG-based recognition of attention state using wavelet and support vector machine,” in 2016 International Seminar on Intelligent Technology and Its Applications (ISITIA) (Lombok: IEEE), 139–144.

[B8] Fujiwara K. Abe E. Kamata K. Nakayama C. Suzuki Y. Yamakawa T. . (2018). Heart rate variability-based driver drowsiness detection and its validation with eeg. IEEE transactions on biomedical engineering 66:1769–1778. doi: 10.1109/TBME.2018.2879346 30403616

[B9] Gupta S. Kumar P. Tekchandani R. (2024). Artificial intelligence based cognitive state prediction in an e-learning environment using multimodal data. Multimedia Tools Appl. 2024, 1–32. doi: 10.1007/s11042-023-18021-x

[B10] Hoglinger M. (2016). ECG preprocessing (Doctoral Dissertation; BS Thesis). Johannes Kepler University Linz, Linz, Austria.

[B11] Hu F. Zhang L. Yang X. Zhang W.-A. (2024). EEG-based driver fatigue detection using spatio-temporal fusion network with brain region partitioning strategy. IEEE Trans. Intellig. Transport. Syst. 25, 9618–9630. doi: 10.1109/TITS.2023.3348517

[B12] Hu H. Wang Z. Zhao X. Li R. Li A. Si Y. . (2024). A survey on brain-computer interface-inspired communications: Opportunities and challenges. IEEE Commun. Surv. Tutorials 27, 108–139. doi: 10.1109/COMST.2024.3396847

[B13] Hu J. Shen L. Sun G. (2018). “Squeeze-and-excitation networks,” in Proceedings of the IEEE Conference on Computer Vision and Pattern Recognition (Salt Lake City, UT: IEEE),7132–7141.

[B14] Huang H. Chen J. Xiao J. Chen D. Zhang J. Pan J. . (2024). Real-time attention regulation and cognitive monitoring using a wearable EEG-based BCI. IEEE Trans. Biomed. Eng. 72, 716–724. doi: 10.1109/TBME.2024.3468351 39320995

[B15] Huang X. Yin E. Wang Y. Saab R. Gao X. (2017). “A mobile eeg system for practical applications,” in 2017 IEEE Global Conference on Signal and Information Processing (GlobalSIP) (Montreal, QC: IEEE), 995–999.

[B16] Jeong D.-H. Jeong J. (2020). In-ear eeg based attention state classification using echo state network. Brain Sci. 10:321. doi: 10.3390/brainsci10060321 32466505 PMC7348757

[B17] Ke Y. Chen L. Fu L. Jia Y. Li P. Zhao X. . (2014). Visual attention recognition based on nonlinear dynamical parameters of eeg. Biomed. Mater. Eng. 24, 349–355. doi: 10.3233/BME-130817 24211916

[B18] Khedher A. B. Jraidi I. Frasson C. (2019). Tracking students' mental engagement using EEG signals during an interaction with a virtual learning environment. J. Intellig. Learn. Syst. Appl. 11, 1–14. doi: 10.4236/jilsa.2019.111001

[B19] Lee D.-H. Kim S.-J. Kim S.-H. (2024). Decoding fatigue levels of pilots using EEG signals with hybrid deep neural networks. arXiv [preprint], (New York, NY: Association for Computing Machinery) arXiv:2411.09707. doi: 10.1109/BCI65088.2025.10931298

[B20] Li Y. Li X. Ratcliffe M. Liu L. Qi Y. Liu Q. (2011). “A real-time eeg-based bci system for attention recognition in ubiquitous environment,” in Proceedings of 2011 International Workshop on Ubiquitous Affective Awareness and Intelligent Interaction (New York, NY: Association for Computing Machinery), 33–40. doi: 10.1145/2030092.2030099

[B21] Lin F.-R. Kao C.-M. (2018). Mental effort detection using eeg data in e-learning contexts. Comp. Educ. 122, 63–79. doi: 10.1016/j.compedu.2018.03.020

[B22] Liu Y. Liu Y. Guo X. Ling X. Geng Q. (2025). Metal surface defect detection using slf-yolo enhanced YOLOv8 model. Sci. Rep. 15:11105. doi: 10.1038/s41598-025-94936-9 40169663 PMC11962121

[B23] Lu Z. Chengao Z. Lu L. Yan Y. Jun W. Wei X. . (2025). Star-YOLO: a lightweight and efficient model for weed detection in cotton fields using advanced YOLOv8 improvements. Comp. Electron. Agricult. 235:110306. doi: 10.1016/j.compag.2025.110306

[B24] Ma X. Dai X. Bai Y. Wang Y. Fu Y. (2024). “Rewrite the stars,” in Proceedings of the IEEE/CVF Conference on Computer Vision and Pattern Recognition (Seattle, WA: IEEE), 5694–5703.

[B25] Nguyen C. D. Minh Q. T. Dinh C. L. Pham N. Q. B. Le Quoc K. Quang L. H. (2022). “Classification of concentration and rest by power spectral analysis with support vector machine model,” in International Conference on the Development of Biomedical Engineering in Vietnam (Cham: Springer), 809–824.

[B26] Niedermeyer E. (2011). Niedermeyer's Electroencephalography: Basic Principles, Clinical Applications, and Related Fields. Philadelphia: Lippincott Williams & Wilkins.

[B27] Peng B. Gao D. Wang M. Zhang Y. (2024). 3d-stcnn: Spatiotemporal convolutional neural network based on eeg 3d features for detecting driving fatigue. J. Data Sci. Intellig. Syst. 2, 1–13. doi: 10.47852/bonviewJDSIS3202983

[B28] Pokhrel S. Chhetri R. (2021). A literature review on impact of covid-19 pandemic on teaching and learning. Higher educ. Future 8, 133–141. doi: 10.1177/2347631120983481

[B29] Salankar N. Koundal D. Chakraborty C. Garg L. (2023). Automated attention deficit classification system from multimodal physiological signals. Multimed. Tools Appl. 82, 4897–4912. doi: 10.1007/s11042-022-12170-1

[B30] Teplan M. (2002). Fundamentals of EEG measurement. Measurem. Sci. Rev. 2, 1–11.

[B31] Toa C. K. Sim K. S. Tan S. C. (2021). Electroencephalogram-based attention level classification using convolution attention memory neural network. IEEE Access 9, 58870–58881. doi: 10.1109/ACCESS.2021.3072731

[B32] van der Linden D. Massar S. A. Schellekens A. F. Ellenbroek B. A. Verkes R.-J. (2006). Disrupted sensorimotor gating due to mental fatigue: preliminary evidence. Int. J. Psychophysiol. 62, 168–174. doi: 10.1016/j.ijpsycho.2006.04.001 16730824

[B33] Wan W. Cui X. Gao Z. Gu Z. (2021). Frontal eeg-based multi-level attention states recognition using dynamical complexity and extreme gradient boosting. Front. Hum. Neurosci. 15:673955. doi: 10.3389/fnhum.2021.673955 34140885 PMC8204057

[B34] Woo S. Park J. Lee J.-Y. Kweon I. S. (2018). “Cbam: Convolutional block attention module,” in Proceedings of the European Conference on Computer Vision (ECCV) (Berlin: Springer), 3–19. doi: 10.1007/978-3-030-01234-2_1

[B35] Wu X. Yang J. Shao Y. Chen X. (2023). Mental fatigue assessment by an arbitrary channel eeg based on morphological features and lstm-cnn. Comput. Biol. Med. 167:107652. doi: 10.1016/j.compbiomed.2023.107652 37950945

[B36] Xu G. Wang Z. Hu H. Zhao X. Li R. Zhou T. . (2024). Riemannian locality preserving method for transfer learning with applications on brain-computer interface. IEEE J. Biomed. Health Informat. 28, 4565–4576. doi: 10.1109/JBHI.2024.3402324 38758616

[B37] Xu G. Wang Z. Zhao X. Li R. Zhou T. Xu T. . (2023). Attentional state classification using amplitude and phase feature extraction method based on filter bank and riemannian manifold. IEEE Trans. Neural Syst. Rehab. Eng. 31, 4402–4412. doi: 10.1109/TNSRE.2023.3329482 37917520

[B38] Xu X. Nie X. Zhang J. Xu T. (2023). Multi-level attention recognition of EEG based on feature selection. Int. J. Environ. Res. Public Health 20:3487. doi: 10.3390/ijerph20043487 36834180 PMC9958593

[B39] Zennifa F. Iramina K. (2019). Quantitative formula of blink rates-pupillometry for attention level detection in supervised machine learning. IEEE Access 7, 96263–96271. doi: 10.1109/ACCESS.2019.2929596

[B40] Zhang M. Luo Z. Xie L. Liu T. Yan Y. Yao D. . (2023). Multimodal vigilance estimation with modality-pairwise contrastive loss. IEEE Trans. Biomed. Eng. 71, 1139–1150. doi: 10.1109/TBME.2023.3328942 37906494

[B41] Zhang Y. Cai H. Nie L. Xu P. Zhao S. Guan C. (2021). An end-to-end 3D convolutional neural network for decoding attentive mental state. Neural Netw. 144, 129–137. doi: 10.1016/j.neunet.2021.08.019 34492547

